# Clinical variants of affective manifestations in the structure of adjustment disorder at forcibly displaced persons as a result of Russia’s invasion of Ukraine

**DOI:** 10.1192/j.eurpsy.2023.1930

**Published:** 2023-07-19

**Authors:** V. Kuzminov, I. Linskiy

**Affiliations:** Urgent psychiatry and Narcology, SI Institute of Neurology Psychiatry and Narcology NAMS of Ukraine, Kharkiv, Ukraine

## Abstract

**Introduction:**

As a result of Russia’s large-scale military aggression against Ukraine, many civilians were forced to leave their homes. Emotional disorders associated with fear for one’s life and relatives, loss of housing, work, and stable social ties are found in the majority of forcibly displaced persons.

**Objectives:**

The purpose of our study was to study the characteristics and expressiveness of affective disorders in displaced persons as a result of Russian aggression against Ukraine. Studied affective disorders in 24 forcibly displaced females. Age of the surveyed 30-50 years. Contacted with psychiatrist because the insistence of relatives. Patients were not identified and applied earlier about mental disorders to psychiatrists. To quantify anxiety, we used a scale HAM-A.

**Methods:**

Clinico-psychopathological.

**Results:**

Affective disorders in the examined patients can be confidently attributed to adjustment disorders. According to the ICD-10, an adaptation disorder (F43.2) was diagnosed. Most of them were dominated by depressive and anxiety-depressive modalities of affective disorders. According to the HAM-A scale, the level of anxiety was 14**±**2.7. Most patients showed few irritability and anger, which is probably due to the inability to respond to the cause of their troubles and problems. However, some patients (6 people) expressed gloomy irritability, anger (clinical sighnificant) a feeling of hostility towards others. It is necessary to note the undulation of these manifestations, often aggravated under the influence of external factors.At the same time, the immediate environment often suffered, on which all the troubles fell. The behavior of such patients became maladaptive, significantly disrupting communication.

**Conclusions:**

Therapeutic impact on internally displaced persons should be aimed not only at overcoming anxiety and depressive syndrome, but also irritability and anger. This should be taken into account when planning psychotherapeutic programs, it is possible to prescribe normotimics.

**Disclosure of Interest:**

None Declared

